# Surgical outcome predictor analysis following hand-assisted or pure laparoscopic transperitoneal nephroureterectomy using the Taiwan upper urinary tract urothelial carcinoma database

**DOI:** 10.3389/fsurg.2022.934355

**Published:** 2022-09-01

**Authors:** Chih-Chun Kuo, Guang-Heng Chen, Chao-Hsiang Chang, Chao-Yuan Huang, Chung-Hsin Chen, Ching-Chia Li, Wen-Jeng Wu, Chih-Chin Yu, Chi-Wen Lo, Yung-Tai Chen, Shin-Hong Chen, Pai-Yu Cheng, Thomas Y. Hsueh, Allen W. Chiu, Po-Han Lin, Jen-Shu Tseng, Jen-Tai Lin, Yuan-Hong Jiang, Chia-Chang Wu, Wei-Yu Lin, Hsu-Che Huang, Han-Sun Chiang, Bing-Juin Chiang

**Affiliations:** ^1^ Department of Urology, Cardinal Tien Hospital, New Taipei City, Taiwan; ^2^ Department of Urology, National Taiwan University Hospital, College of Medicine, National Taiwan University, Taipei, Taiwan; ^3^ Department of Urology, China Medical University and Hospital, Taichung, Taiwan; ^4^ Department of Urology, China Medical University Hsinchu Hospital, Hsinchu, Taiwan; ^5^ School of Medicine, China Medical University, Taichung, Taiwan; ^6^ Department of Urology, Kaohsiung Medical University Hospital, Kaohsiung, Taiwan; ^7^ Department of Urology, School of Medicine, College of Medicine, Kaohsiung Medical University, Kaohsiung, Taiwan; ^8^ Graduate Institute of Clinical Medicine, College of Medicine, Kaohsiung Medical University, Kaohsiung, Taiwan; ^9^ Division of Urology, Department of Surgery, Taipei Tzu Chi Hospital, The Buddhist Tzu Chi Medical Foundation, New Taipei City, Taiwan; ^10^ School of Medicine, Buddhist Tzu Chi University, Hualien, Taiwan; ^11^ Department of Urology, Taiwan Adventist Hospital, Taipei, Taiwan; ^12^ Division of Urology, Department of Surgery, Far-Eastern Memorial Hospital, New Taipei, Taiwan; ^13^ Division of Urology, Department of Surgery, Taipei City Hospital Ren-Ai Branch, Taipei, Taiwan; ^14^ Department of Urology, School of Medicine, National Yang Ming Chiao Tung University, Taipei, Taiwan; ^15^ College of Medicine, National Yang Ming Chiao Tung University, Taipei, Taiwan; ^16^ Department of Urology, MacKay Memorial Hospital, Taipei, Taiwan; ^17^ Mackay Medical College, Taipei, Taiwan; ^18^ Institute of Biomedical Informatics, National Yang Ming Chiao Tung University, Taipei, Taiwan; ^19^ Division of Urology, Department of Surgery, Kaohsiung Veterans General Hospital, Kaohsiung, Taiwan; ^20^ Department of Urology, Hualien Tzu Chi Hospital, Buddhist Tzu Chi Medical Foundation and Tzu Chi University, Hualien, Taiwan; ^21^ Department of Urology, Shuang Ho Hospital, Taipei Medical University, New Taipei City, Taiwan; ^22^ Department of Urology, School of Medicine, College of Medicine, Taipei Medical University, Taipei, Taiwan; ^23^ TMU Research Center of Urology and Kidney, Taipei Medical University, Taipei, Taiwan; ^24^ Division of Urology, Department of Surgery, Chang Gung Memorial Hospital, Chia-Yi, Taiwan; ^25^ Chang Gung University of Science and Technology, Chia-Yi, Taiwan; ^26^ Department of Medicine, Chang Gung University, Taoyuan, Taiwan; ^27^ Department of Life Science, College of Science, National Taiwan Normal University, Taipei, Taiwan; ^28^ Department of Urology, Fu Jen Catholic University Hospital, Fu Jen Catholic University, New Taipei City, Taiwan; ^29^ School of Medicine, Fu-Jen Catholic University, New Taipei City, Taiwan

**Keywords:** urothelial carcinoma, upper urinary tract urothelial carcinoma, laparoscopic nephroureterectomy, hand-assisted laparoscopic nephroureterectomy, oncological outcome, surgical volume

## Abstract

**Purpose:**

Taiwan has a high incidence of upper tract urothelial carcinoma (UTUC). This study aimed to compare the surgical outcomes following transperitoneal hand-assisted laparoscopic nephroureterectomy (TP-HALNU) and transperitoneal pure laparoscopic nephroureterectomy (TP-LNU) from the Taiwan nationwide UTUC collaboration database using different parameters, including surgical volumes.

**Materials and methods:**

The nationwide UTUC collaboration database includes 14 hospitals in Taiwan from the Taiwan Cancer Registry. We retrospectively reviewed the records of 622 patients who underwent laparoscopic nephroureterectomy between July 1988 and September 2020. In total, 322 patients who received TP-LNU or TP-HALNU were included in the final analysis. Clinical and pathological data and oncological outcomes were compared.

**Results:**

Of the 322 patients, 181 and 141 received TP-LNU and TP-HALNU, respectively. There were no differences in clinical and histopathological data between the two groups. No differences were observed in perioperative and postoperative complications. There were no significant differences in oncological outcomes between the two surgical approaches. In the multivariate analysis, the cohort showed that age ≥70 years, positive pathological lymph node metastasis, tumors located in the upper ureter, and male sex were predictive factors associated with an increased risk of adverse oncological outcomes. A surgical volume of ≥20 cases showed a trend toward favorable outcomes on cancer-specific survival [hazard ratio (HR) 0.154, *p* = 0.052] and marginal benefit for overall survival (HR 0.326, *p* = 0.019) in the multivariate analysis.

**Conclusion:**

Although different approaches to transperitoneal laparoscopic nephroureterectomy showed no significant differences in surgical outcomes, age, sex, lymph node metastasis, and tumor in the upper ureter in the following period were predictive factors for oncological outcomes. Higher surgical volume did not impact disease-free survival and bladder recurrence-free survival but was associated with improved overall survival and cancer-specific survival. Exploration of unknown influencing factors is warranted.

## Introduction

Urothelial carcinoma (UC) is the fourth most common malignancy worldwide, with upper tract UC (UTUC) accounting for 5%–10% of these cases (1, 2). The gold standard for UTUC management is radical nephroureterectomy (NU), with the removal of the ipsilateral bladder cuff (2, 3). This procedure has historically been performed *via* an open approach [open NU (ONU)]; however, concerns regarding associated perioperative morbidity have led to the widespread adoption of minimally invasive surgery, laparoscopic NU (LNU), and robot-assisted NU (4–7).

LNU was first reported in 1991 (8). Its widespread adoption was initially limited by technical challenges and concerns about tumor cell dissemination *via* pneumoperitoneum (9), and it was later demonstrated that there was no difference in the risk of local recurrence between ONU and LNU (10). Minimally invasive NU results in shorter hospital stays, less blood loss, fewer complications, decreased use of postoperative pain medications, and superior patient satisfaction (5, 6, 9, 11–13). Most importantly, several multicenter retrospective studies, randomized trials, and systematic reviews have evaluated the oncological outcomes of ONU vs. LNU and have demonstrated equivalent oncological outcomes (4–7, 11, 13–16). It is not surprising, therefore, that for the exclusion of invasive tumors, larger tumors, or metastatic tumors, the use of LNU has increased in recent years (17).

However, the increased operation time, steep learning curve, and need for highly experienced laparoscopic surgeons have limited its widespread use. Hand-assisted laparoscopy (HAL) has provided a new minimally invasive alternative for patients with UTUC (18). HAL uses a unique approach that combines the finest aspects of open and laparoscopic surgery and *en bloc* specimen retrieval, thus maintaining the oncological principles used in open surgery (19).

LNU and hand-assisted LNU (HALNU) had comparable oncological and better perioperative and postoperative outcomes than ONU, but HALNU may be inferior to LNU or ONU in terms of recurrence-free survival and intravesical recurrence-free survival rates (20, 21). However, these studies are heterogeneous as they encompassed transperitoneal or retroperitoneal approaches. Some studies showed different oncological results with either approach (22). Considering the limited number of laparoscopic retroperitoneal nephroureterectomy cases in Taiwan, we conducted a study focusing on the surgical outcomes following transperitoneal HALNU (TP-HALNU) or transperitoneal pure LNU (TP-LNU) using the Taiwan nationwide UTUC collaboration database.

In Taiwan, the training of urology residents includes laparoscopic surgery in individual secondary or tertiary referral hospitals. Residents must pass their own training programs through direct observation of procedural skills. However, hands-on laparoscopic procedures have not been tested in the national licensure examination. Laparoscopic procedures are not restricted to subspecialists. Surgical volumes and methods of individualized centers may considerably vary. Several studies have revealed that the learning curve may vary among individual surgeons, and a consensus should be reached for the minimum number of cases to achieve proficiency (23). Surgical volumes also affect outcomes (24). The learning thresholds with fewer intraoperative and perioperative complications varied in the literature (25, 26). A recent study found longer operation time and a trend toward more complications with <20 cases (27). In addition, a higher surgical volume (≥20 cases) of laparoscopic hysterectomy was inversely related to the conversion rate to laparotomy (28). Although the length of the learning curve has individual differences, low surgical volumes may impact surgical outcomes. Hence, the surgical volume (≥20 cases or not) was taken into evaluation in this Taiwan UTUC collaboration study.

## Materials and methods

The Taiwan Cancer Registry (TCR), a population-based cancer registry, is a nationwide cancer registry. Hospitals with >50-bed capacity that provide outpatient and hospitalized cancer care were recruited to participate in reporting all newly diagnosed malignant neoplasms to the registry. The TCR was queried for registered patients diagnosed with malignant neoplasms of the renal pelvis and ureter between July 1988 and October 2020. The involved hospitals are listed in Supplementary Table S1.

All patients with a primary diagnosis of renal pelvic or ureteral neoplasm who underwent NU were identified using the International Classification of Diseases Ninth Revision diagnostic and procedure codes. Exclusion criteria included patients who did not undergo NU, who did not receive laparoscopic or HAL surgery, who had previous or synchronous bladder UC, who received neoadjuvant or adjuvant chemotherapy, and who underwent retroperitoneal laparoscopic surgery, as well as histological type other than UC, bilateral neoplasm, or graft neoplasm.

We retrospectively reviewed patient records from the Urology Research Study Group database of 14 participating Taiwanese hospitals. The database included patients with UTUC recorded between July 1988 and September 2020, of whom 322 patients who underwent LNU between June 2002 and August 2019 were selected for this study. This study was approved by the institutional review boards of our hospitals (CTH107-3-5-035 and 06-X34-105).

The demographic data included age and sex. The distribution of patients between the two surgical approaches was recorded. Tumor characteristics, including tumor size, tumor location, laterality, multiplicity, histological characteristics, surgical margin, and pathological staging, were recorded. Outcome assessments included complications, mortality, and a disease-free period. Complications included surgical complications recorded according to the Clavien–Dindo classification and postoperative complications, including ileus and ventral hernia. We also divided patients by the surgical volume of hospitals, with the higher volume group comprising all patients from hospitals with ≥20 nephroureterectomy cases and the lower volume group comprising patients from hospitals with <20 cases. Survival outcome parameters were defined as all-cause death, cancer-specific survival (CSS) as death due to UTUC, disease-free survival (DFS) as cancer recurrence or metastasis, and bladder recurrence-free survival (BRFS) as UC recurrence in the bladder.

Statistical analyses were performed using SPSS Statistics (IBM, Armonk, NY, USA, version 24). The Cox proportional hazard model was selected to assess the effect of the surgical approach on prognostic outcomes, alone and after adjusting for potential confounders. All parameters were analyzed using univariate analysis, and those of specific interest were included in the multivariate analysis. All statistical assessments were two-tailed and considered statistically significant at *p* < 0.05.

## Results

**Figure 1:**
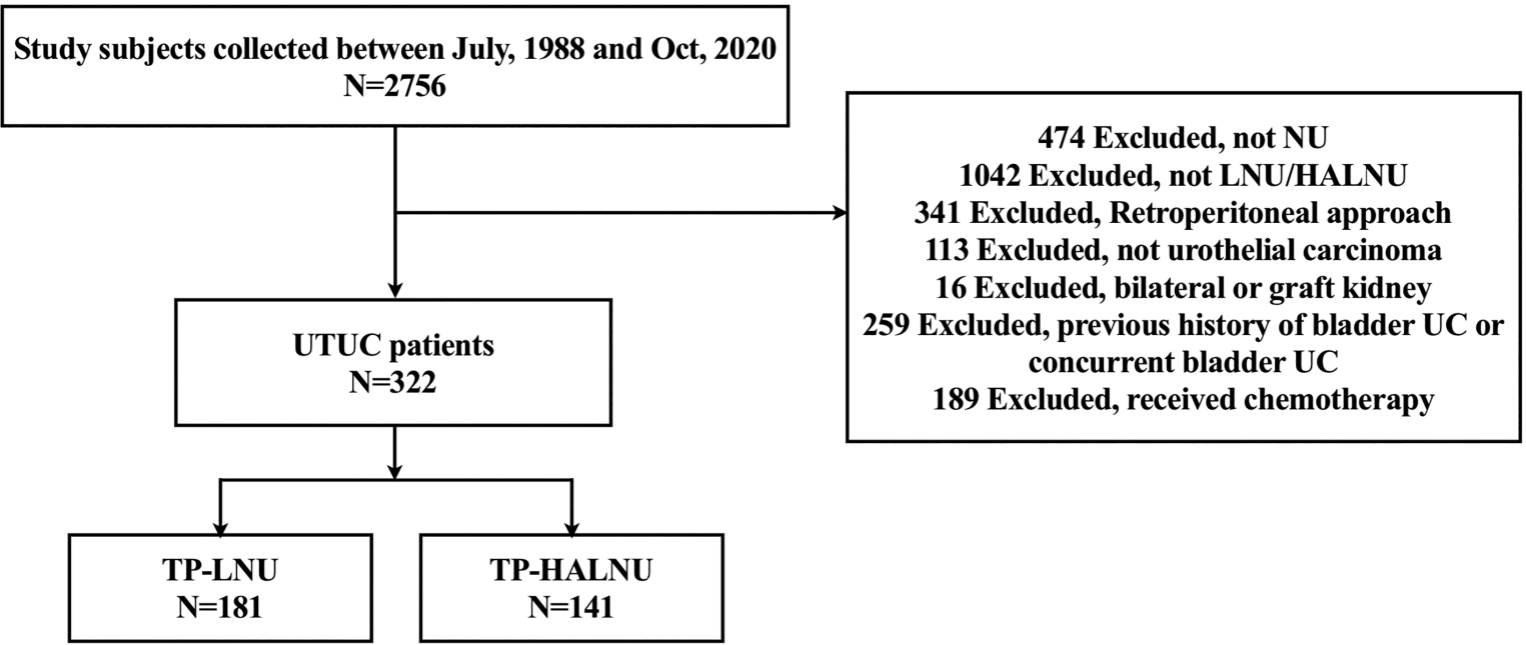
Study flowchart. UTC, upper tract urothelial carcinoma; UC, urothelial carcinoma; NU, nephroureterectomy; LNU, laparoscopic nephroureterectomy; HALNU, hand-assisted laparoscopic nephroureterectomy; TP-LNU, transperitoneal laparoscopic nephroureterectomy; TP-HALNU, transperitoneal hand-assisted laparoscopic nephroureterectomy.

In total, 663 patients who underwent LNU were included in this study. The patients were divided into four different surgical approach groups: TP-LNU, TP-HALNU, retroperitoneal LNU, and retroperitoneal HALNU, with 181, 141, 41, and 300 patients in each group, respectively (Figure 1). The transperitoneal group was enrolled, and 322 patients were enrolled in the final analyses.

Patient demographic and disease-specific characteristics are shown in Table 1. No significant difference in age was noted between these two groups, and the hand-assisted group had more male patients. There were no significant differences in tumor size, laterality, or multiplicity. We noted no significant difference in perioperative complications according to the Clavien–Dindo classification and postoperative complications of ileus and ventral herniation. TP-HALNU showed a higher overall mortality rate (44.7% vs. 26%) in the TP-LNU group but not in surgery-related mortality. Patients with TP-HALNU had a higher percentage of bladder UC cases after NU. The follow-up period for each approach was 30.26 months for TP-LNU and 62.06 months for TP-HALNU.

**Table 1:** Demographic data of UTUC patients.

Variables	TP-LNU (*N* = 181)		TP-HALNU (*N* = 141)		*p*-value
	*N*	%	*N*	%	
Sex					
Male	58	32.0	57	40.4	0.019
Female	123	68.0	84	59.6	
Age (mean ± SD)	69.46 ± 10.8		69.62 ± 9.77		
Tumor size					
<1 cm	15	8.5	9	6.4	0.416
≥1 and <2 cm	53	30.1	39	27.7	
≥2 and <3 cm	44	25.0	29	20.6	
≥3 cm	63	35.8	61	43.3	
Laterality					
Left	91	50.3	63	44.7	0.319
Right	90	49.7	78	55.3	
Multiplicity					
No	148	85.1	95	67.4	<0.001
Yes	26	14.9	46	32.6	
Clavien–Dindo classification					
No	123	68.7	105	75.5	0.131
Grade I	26	14.5	7	5.0	
Grade II	21	11.7	20	14.4	
Grade III	5	2.8	5	3.6	
Grade IV	3	1.7	1	0.7	
Grade V	1	0.6	1	0.7	
Post-OP complication					
Ileus	2	1.1	1	0.7	0.731
Ventral hernia	3	1.7	2	1.5	0.887
Mortality					
No	134	74.0	78	55.3	0.008
UTUC related	3	1.7	8	5.7	
Non-UTUC related	11	6.1	12	8.5	
Unknown	32	17.7	42	29.8	
Surgery related	1	0.6	1	0.7	
Follow-up (months) median	30.26		62.06		

UTUC, upper tract urothelial carcinoma; NU, nephroureterectomy; TP-LNU, transperitoneal laparoscopic NU; TP-HALNU, transperitoneal hand-assisted laparoscopic NU; pNx, regional lymph nodes cannot be assessed; UC, urothelial carcinoma.

Detailed tumor characteristics were also recorded, which showed no significant differences in tumor location, surgical margin status, or pathological staging (Table 2). TP-HALNU had more cases of high-grade tumors, less pathologically positive lymph nodes, and more bladder UC after NU.

**Table 2:** Tumor characteristics of UTUC patients.

Variables	TP-LNU (*N* = 181)		TP-HALNU (*N* = 141)		*p-*value
	*N*	%	*N*	%	
Tumor location					
Renal pelvis	111	61.3	99	70.2	0.097
Upper ureter	38	21.0	31	22.0	0.830
Middle ureter	19	10.5	16	11.3	0.808
Lower ureter	29	16.0	25	17.7	0.684
Bladder cuff	1	0.6	2	1.4	0.422
NU histology					
Low grade	39	22.0	28	20.0	0.003
High grade	138	78.0	112	80.0	
Surgical margin					
Free	178	98.3	137	97.9	0.750
Positive	3	1.7	3	2.1	
Pathological stage T					
pTis	3	1.7	5	3.6	0.601
pTa	52	28.7	32	22.9	
pT0	3	1.7	1	0.7	
pT1	49	27.1	46	32.9	
pT2	35	19.3	30	21.4	
pT3	36	19.9	25	17.9	
pT4	3	1.7	1	0.7	
Pathological stage N					
pN0	64	35.4	13	9.3	< 0.001
pN+	3	1.7	1	0.7	
pNx	114	63.0	126	90.0	
Bladder UC after NU					
No	135	77.1	95	67.9	0.045
Yes	40	22.9	45	32.1	

UTUC, upper tract urothelial carcinoma; NU, nephroureterectomy; TP-LNU, transperitoneal laparoscopic NU; TP-HALNU, transperitoneal hand-assisted laparoscopic NU; pNx, regional lymph nodes cannot be assessed; UC, urothelial carcinoma.

In the univariate survival analysis, surgical approach, histologic grade, tumor size, tumor laterality, and tumor location in the renal pelvis, middle ureter, lower ureter, and bladder cuff were independent predictors of survival outcomes (Table 3). The female sex was associated with shorter BRFS, with a hazard ratio (HR) of 0.478 (*p* = 0.001), but was not associated with other survival parameters. Age >70 years was independently associated with poorer overall survival (OS), CSS, and DFS [HR 3.324 (*p* = 0.001), 6.290 (*p* = 0.017), and 3.029 (*p* = 0.005), respectively]. Advanced pathological T staging and positive pathological N staging were associated with survival outcomes, with pT3 staging showing HR of 10.911 (*p* = 0.027) and 3.098 (*p* = 0.029) for CSS and DFS, and pN+ staging showing HR of 17.279 (*p* = 0.011) and 10.961 (*p* = 0.031) for OS and DFS, respectively. Tumor multiplicity was associated with DFS and BRFS, with HR of 3.446 (*p* < 0.001) and 1.784 (*p* = 0.014), respectively. Analysis of tumor location showed that only tumors located in the upper ureter were associated with DFS (HR 2.054, *p* = 0.047). Higher surgical volume (>20 cases) was associated with OS, with an HR of 0.425 (*p* = 0.046).

**Table 3:** Comparative univariate survival analysis of UTUC patients.

Univariate analysis		OS		CSS		DFS		BRFS	
		HR (95% CI)	*p*-value	HR (95% CI)	*p-*value	HR (95% CI)	*p*-value	HR (95% CI)	*p*-value
Approach	TP-LNU	1		1		1		1	
	TP-HALNU	0.854 (0.423, 1.724)	0.659	1.931 (0.576, 6.466)	0.286	1.540 (0.744, 3.184)	0.244	1.146 (0.743, 1.767)	0.537
Sex	Male	1		1		1		1	
	Female	0.897 (0.460, 1.749)	0.750	0.519 (0.174, 1.550)	0.240	0.821 0.411, 1.641)	0.577	0.478 (0.312, 0.732)	0.001
Age	<70	1		1		1		1	
	≥70	3.324 (1.597, 6.919)	0.001	6.290 (1.389,28.477)	0.017	3.029 (1.405, 6.529)	0.005	1.518 (0.988, 2.331)	0.057
NU histology	Low grade	1		1		1		1	
	High grade	1.062 (0.497, 2.272)	0.876	1.843 (0.408, 8.321)	0.427	1.861 (0.716, 4.835)	0.203	0.829 (0.507, 1.356)	0.455
pT stage	pTis/pTa/pT0	1		1		1		1	
	pT1	0.628 (0.268, 1.471)	0.285	1.886 (0.171, 20.818)	0.604	1.103 (0.370, 3.287)	0.860	0.932 (0.523, 1.663)	0.813
	pT2	0.576 (0.205, 1.617)	0.295	5.954 (0.665, 53.284)	0.111	2.491 (0.905, 6.857)	0.077	1.310 (0.717, 2.391)	0.380
	pT3	1.328 (0.566, 3.117)	0.514	10.911 (1.312, 90.732)	0.027	3.098 (1.125, 8.531)	0.029	1.770 (0.976, 3.209)	0.060
	pT4	—	0.983	—	0.991	—	0.981	—	0.960
pN stage	pN0	1		1		1		1	
	pN+	17.279 (1.896, 157.5)	0.011	—	0.944	10.916 (1.250, 95.361)	0.031	—	0.964
Tumor size	<1 cm	1		1		1		1	
	≥1 and <2 cm	1.313 (0.291, 5.933)	0.724	—	0.943	1.074 (0.120, 9.608)	0.949	1.814 (0.631, 5.212)	0.269
	≥2 and <3 cm	0.867 (0.173, 4.330)	0.861	—	0.942	2.069 (0.248, 17.233)	0.501	1.737 (0.587, 5.143)	0.319
	≥3 cm	1.380 (0.314, 6.067)	0.670	—	0.929	4.409 (0.592, 32.815)	0.147	2.204 (0.785, 6.186)	0.134
Multiplicity	No	1		1		1		1	
	Yes	1.770 (0.883, 3.549)	0.108	1.509 (0.465, 4.902)	0.493	3.446 (1.741, 6.822)	<0.001	1.784 (1.124, 2.832)	0.014
Laterality	Left	1		1		1		1	
	Right	1.969 (0.984, 3.941)	0.056	1.114 (0.374, 3.318)	0.846	0.875 (0.442, 1.732)	0.701	0.744 (0.485, 1.141)	0.175
Renal pelvis	No	1		1		1		1	
	Yes	0.922 (0.466, 1.822)	0.815	2.882 (0.638, 13.020)	0.169	1.678 (0.756, 3.722)	0.203	1.451 (0.905, 2.328)	0.122
Upper ureter	No	1		1		1		1	
	Yes	1.358 (0.654, 2.821)	0.411	1.557 (0.479, 5.060)	0.461	2.054 (1.010, 4.176)	0.047	1.515 (0.939, 2.446)	0.089
Middle ureter	No	1		1		1		1	
	Yes	1.510 (0.586, 3.895)	0.394	0.042 (0.000, –)	0.431	0.554 (0.133, 2.318)	0.419	0.470 (0.190, 1.161)	0.102
Lower ureter	No	1		1		1		1	
	Yes	1.133 (0.496, 2.589)	0.766	0.903 (0.200, 4.075)	0.894	1.130 (0.466, 2.739)	0.786	1.151 (0.659, 2.011)	0.622
Bladder cuff	No	1		1		1		1	
	Yes	0.049	0.803	0.049	0.849	4.973 (0.668, 37.009)	0.117	3.313 (0.812, 13.518)	0.095
Surgical volume	Lower (<20)	1		1		1		1	
	Higher (≥20)	0.425 (0.183, 0.985)	0.046	0.366 (0.080, 1.679)	0.196	1.042 (0.502, 2.163)	0.912	1.146 (0.727, 1.806)	0.556

UTUC, upper tract urothelial carcinoma; OS, overall survival; CSS, cancer-specific survival; DFS, disease-free survival; BRFS, bladder recurrence-free survival; HR, hazard ratio; Cl, confidence interval; TP-LNU, transperitoneal laparoscopic nephroureterectomy; TP-HALNU, transperitoneal hand-assisted laparoscopic nephroureterectomy; UTUC, upper tract urothelial carcinoma; OS, overall survival; CSS, cancer-specific survival; DFS, disease-free survival; BRFS, bladder recurrence-free survival; HR, hazard ratio; Cl, confidence interval.

In the multivariate survival analysis, surgical approach, pathological T staging, tumor multiplicity, and tumor location in the renal pelvis, middle ureter, lower ureter, or bladder cuff showed no association with survival outcomes (Table 4). The female sex was associated with BRFS, with an HR of 0.421 (*p* < 0.001), but was not associated with other survival parameters. Age >70 years was associated with all four parameters except BRFS, with HR values of 4.146 (*p* = 0.001), 20.310 (*p* = 0.008), and 3.349 (*p* = 0.005), respectively. Positive pathological N staging was associated with OS and DFS, with HR values of 88.379 (*p* < 0.001) and 34.717 (*p* = 0.005), respectively. Analysis of tumor location showed that only tumors in the upper ureter were associated with BRFS, with an HR of 2.537 (*p* = 0.034). Higher surgical volume showed a trend toward better CSS (*p* = 0.052) and was associated with OS (HR 0.326, *p* = 0.019).

**Table 4:** Comparative multivariable survival analysis of UTUC patients.

Multivariable analysis		OS		CSS		DFS		BRFS	
		HR (95% CI)	*p*-value	HR (95% CI)	*p*-value	HR (95% CI)	*p*-value	HR (95% CI)	*p*-value
Approach	TP-LPN	1		1		1		1	
	TP-HALPN	0.922 (0.424–2.007)	0.838	1.582 (0.432–5.786)	0.488	1.085 (0.478–2.464)	0.845	0.989 (0.614–1.593)	0.965
Sex	Male	1		1		1		1	
	Female	0.649 (0.316–1.333)	0.239	0.319 (0.091–1.114)	0.073	0.650 (0.303–1.392)	0.267	0.421 (0.268–0.662)	<0.001
Age	<70	1		1		1		1	
	≥70	4.146 (1.849–9.295)	0.001	20.310 (2.220–185.77)	0.008	3.439 (1.452–8.141)	0.005	1.561 (0.972–2.506)	0.065
pT stage	pTis/pTa/pT0	1		1		1		1	
	pT1	0.472 (0.192–1.162)	0.103	1.233 (0.108–14.022)	0.866	0.825 (0.269–2.537)	0.738	0.765 (0.417–1.402)	0.386
	pT2	0.462 (0.158–1.351)	0.158	7.921 (0.818–76.706)	0.074	2.365 (0.830–6.741)	0.107	1.480 (0.784–2.792)	0.227
	pT3	0.804 (0.309–2.092)	0.655	6.296 (0.684–57.942)	0.104	1.754 (0.569–5.409)	0.328	1.317 (0.689–2.518)	0.405
pN stage	pN0	1		1		1		1	
	pN+	88.379 (7.284–1072)	<0.001	21,117,611.8 (—)	0.935	34.717 (2.912–413.89)	0.005	0.000 (0.000)	0.983
Multiplicity	No	1		1		1		1	
	Yes	1.731 (0.561–5.336)	0.340	0.710 (0.046–11.004)	0.806	2.644 (0.850–8.221)	0.093	1.026 (0.459–2.290)	0.950
Renal pelvis	No	1		1		1		1	
	Yes	0.621 (0.157–2.467)	0.499	2.198 (0.135–35.918)	0.580	1.219 (0.286–5.192)	0.789	2.473 (0.925–6.612)	0.071
Upper ureter	No	1		1		1		1	
	Yes	0.813 (0.230–2.872)	0.747	1.123 (0.083–15.245)	0.930	1.131 (0.355–3.608)	0.835	2.537 (1.074–5.992)	0.034
Middle ureter	No	1		1		1		1	
	Yes	1.017 (0.190–5.433)	0.985	0.000 (—)	0.968	0.547 (0.080–3.744)	0.539	0.930 (0.267–3.241)	0.909
Lower ureter	No	1		1		1		1	
	Yes	0.647 (0.145–2.880)	0.567	0.946 (0.073–12.344)	0.966	0.922 (0.228–3.720)	0.909	2.166 (0.785–5.978)	0.136
Bladder cuff	No	1		1		1		1	
	Yes	0.000 (—)	0.993	0.000 (—)	0.996	5.985 (0.548–65.409)	0.143	4.372 (0.864–22.120)	0.074
Surgical volume	Lower (<20)	1		1		1		1	
	Higher (≥20)	0.326 (0.128–0.831)	0.019	0.154 (0.023–1.019)	0.052	1.025 (0.460–2.284)	0.952	1.211 (0.745–1.969)	0.439

UTUC, upper tract urothelial carcinoma; OS, overall survival; CSS, cancer-specific survival; DFS, disease-free survival; BRFS, bladder recurrence-free survival; HR, hazard ratio; Cl, confidence interval; TP-LNU, transperitoneal laparoscopic nephroureterectomy; TP-HALNU, transperitoneal hand-assisted laparoscopic nephroureterectomy.

## Discussion

The present study aimed to compare the surgical outcomes following TP-HALNU and TP-LNU from the Taiwan nationwide UTUC collaboration database for different parameters, including surgical volumes. We compared surgical outcomes, including OS, CSS, DFS, and BRFS, between the two surgical approaches in a nationwide database. Our study showed that the choice of surgical approach varies among urology departments or surgeon preferences, which shows a significant divergence between hospitals.

We noticed a significant difference in mortality between the two surgical approaches, with the TP-HALNU approach having an overall mortality rate of 44.7% compared to 26% in TP-LNU. This significant difference in overall mortality might be caused by the different follow-up periods (62 vs. 30 months). Although the two approaches showed significant differences in overall mortality, after the univariate and multivariate analyses for oncological outcomes, TP-LNU and TP-HALNU were not oncological predictors. Surgery-related mortality showed no difference, with a mortality rate of 0.7% vs. 0.6% between the two groups. The most common cause of death was listed as not UTUC-related. However, this was a cross-sectional, large-scale, retrospective cohort study using the UTUC collaboration database of the Taiwan Urology Association. Despite the presence of limitations, the results represent real-world data and demonstrate that either TP-LNU or TP-HALNU is feasible in the current setting in Taiwan. In addition, perioperative complications, according to the Clavien–Dindo classification, showed no significant difference between the two approaches.

Several previous studies comparing HALNU with LNU or ONU showed comparable oncological outcomes and better perioperative and postoperative outcomes (20, 21, 29–32). However, HALNU may be inferior to LNU or ONU with respect to RFS and BRFS rates (20, 33) and may be associated with higher intravesical recurrence (32). The current study demonstrated no oncological outcome difference between TP-LNU and TP-HALNU in both univariate and multivariate analyses.

In this study, we concluded that age was an independent predictor of survival outcomes. The multivariate analysis showed an association between age and OS, CSS, and DFS, with an increased risk in patients aged ≥70 years. Another independent predictor was sex, which was a lower risk factor in female patients with BRFS. This finding is consistent with previous reports illustrating that male sex was strongly associated with intravesical recurrence in patients with UTUC who received radical NU (34–36). Other than BRFS, no significant association was noted between sex and survival outcomes. Another obvious independent predictor associated with oncological outcomes was positive pathological lymph node metastasis, which is an obvious risk factor associated with worse outcomes.

NU with bladder cuff excision is the gold standard treatment for UTUC (37–39), so tumors located at the bladder cuff present challenges for radical resection. There are several approaches to bladder cuff excision, including the open technique, transurethral incision of the ureteral orifice, intussusception technique, transvesical laparoscopic detachment, and laparoscopic stapling (40, 41). In our study, tumor location at the bladder cuff was not associated with oncological outcomes. However, this database did not provide detailed records of the bladder cuff excision methods or the margin status of the bladder cuff. This could be a possible confounding factor, although one large patient cohort in Taiwan concluded that the method by which the bladder cuff is removed does not affect cancer-specific outcomes (42).

Retroperitoneoscopic NU (RPNU), with or without hand assistance, is also a widely accepted treatment option for UTUC. Previous studies have shown that RPNU had comparable oncological outcomes compared with ONU (43), LNU (44), or HALNU (45), and may have better perioperative and postoperative outcomes than LNU (44). However, intestinal retraction is considerably easier with the transperitoneal approach. Besides, peritoneal tear during RPNU occurred in certain cases, even with experienced surgeons (46). The impact of peritoneal tear includes conversion to the transperitoneal approach, but the limited number of retroperitoneal LNU cases in the Taiwan UTUC database may confound the analysis. Thus, in this study, we compared TP-HALNU and TP-LNU and observed comparable outcomes. We suggest that either approach is safe and feasible, depending on surgeons’ preferences and experiences.

For surgical volume, we observed that higher surgical volume did not impact DFS or BRFS but was associated with a trend toward favorable CSS (HR 0.154, *p* = 0.052) and marginal benefit for OS (HR 0.326, *p* = 0.019). Several previous studies have reported fewer intraoperative and perioperative complications associated with the surgical learning curve, reporting different learning thresholds at 50 or 100 cases at a single center (25, 26). In the current study, lower surgical volume (<20 cases) did not significantly influence DFS or BRFS. However, higher surgical volume was associated with a trend toward improved CSS (*p* = 0.052). Although this result implies that surgical volume matters in oncological outcomes, it is difficult to conclude that lower surgical volume impacts OS by influencing the quality of oncological control in the results. A poor correlation between PFS and OS may occur when considering different tumor characteristics, recurrence patterns, subsequent heterogeneous treatment, and quality of care, which were not shown in this study (47).

From this study, we suggest that either method is safe and feasible, depending on the surgeon's preferences and experiences. Larger-scale and prospective studies are required, considering the surgical volume and learning curve.

## Limitations

The current study had several limitations. First, the original database included retrospectively reviewed patients with a lack of detailed records on several parameters, including bladder cuff resection method, margin status of the bladder cuff, the extent of lymph node dissection, perioperative intravesical chemotherapy, and behavioral adjustments such as smoking discontinuation. All mortality data were retrieved from the National Cancer Registry, and patients without an assigned code for cause of death were grouped into “Unknown” in the mortality parameter. This could have caused a statistical bias for CCS. The current study enrolled 14 hospitals in which patients were not randomized for the two surgical approaches. The choice of approach, the extent of lymph node dissection, and the follow-up protocol were mainly decided by each surgeon's or urology department's preference, which may cause bias in comparing outcomes between different approaches.

## Conclusion

No significant differences in oncological outcomes and postoperative complication rates were observed between the TP-LNU and TP-HALNU groups. Age, sex, and lymph node metastasis were independent predictors of oncological outcomes. Higher surgical volume did not impact DFS and BRFS but was associated with improved OS and CSS. Exploration of unknown influencing factors is warranted.

## Data Availability

The raw data supporting the conclusions of this article will be made available by the authors, without undue reservation.
