# Supplementary material for: Surgical outcome predictor analysis following hand-assisted or pure laparoscopic transperitoneal nephroureterectomy using the Taiwan upper urinary tract urothelial carcinoma database
Source: Front Surg. 2022 Sep 1;9:934355. doi: 10.3389/fsurg.2022.934355 (PMC9475171; doi:10.3389/fsurg.2022.934355)
Supplement: Supplementary file 1 [file Table_1_v1.docx]

Supplementary Table 1 Surgery case distribution

| Variables | TP-LNU  (N=181) | | TP-HALNU (N=141) | |
| --- | --- | --- | --- | --- |
|  | N | % | N | % |
| Hospital |  |  |  |  |
| Taipei Tzu Chi Hospital | 8 | (4.4) | 5 | (3.5) |
| Hualien Tzu Chi Hospital | 9 | (5.0) | 1 | (0.7) |
| Kaoshiung Medical University Hospital | 2 | (1.1) | 5 | (3.5) |
| Chang Gung Memorial Hospital ,Chiayi | 1 | (0.6) | 0 | (0.0) |
| Kaohsiung Veterans General Hospital | 12 | (6.6) | 1 | (0.7) |
| China Medical University Hospital | 125 | (69.1) | 1 | (0.7) |
| Taipei City Hospital | 0 | (0.0) | 0 | (0.0) |
| National Taiwan University Hospital | 1 | (0.6) | 73 | (51.8) |
| Taipei Medical University-Shuang Ho Hospital | 8 | (4.4) | 0 | (0.0) |
| Taiwan Adventist Hospital | 1 | (0.6) | 4 | (2.8) |
| Cardinal Tien Hospital | 2 | (1.1) | 3 | (2.1) |
| Far Eastern Memorial Hospital | 9 | (5.0) | 24 | (17.0) |
| Taipei Medical University Hospital | 3 | (1.7) | 23 | (16.3) |
| Mackay Memorial Hospital | 0 | (0.0) | 1 | (0.7) |

TP-LNU: transperitoneal laparoscopic nephroureterectomy, TP-HALNU: transperitoneal hand-assisted laparoscopic nephroureterectomy
